# A Survey on Enhanced Recovery After Surgery (ERAS) Elements in Cleft Palate Repair

**DOI:** 10.1177/10556656221103756

**Published:** 2022-05-26

**Authors:** Christina Grabar, Jennifer Fligor, Melissa Kanack, Juleah Walsh, Joe Kim, Raj Vyas

**Affiliations:** 1 12219 School of Medicine, University of California Irvine, Orange, CA, USA; 2 Department of Plastic Surgery, 8788 University of California Irvine, Orange, CA, USA; 3 Pediatric Plastic Surgery, 20209 CHOC Children’s, Orange, CA, USA

**Keywords:** palatoplasty, surgical complications, speech development

## Abstract

**Objective:**

This study aims to characterize current use, knowledge, and attitude toward ERAS protocols by academic craniofacial surgeons.

**Design:**

Craniofacial surgeons were provided with electronic surveys.

**Setting:**

Electronic survey; Institutional tertiary surgeons.

**Participants:**

102 cleft palate surgeons surveyed and 31 completed the survey (30.4%).

**Interventions:**

None.

**Main Outcome Measures:**

Respondents rated their knowledge, use, and willingness to implement perioperative interventions modeled after adult ERAS protocols.

**Results:**

Majority (67.7%) rated they were knowledgeable about ERAS. However, 61.3% “never use” a standardized protocol for cleft palate surgery. Only 3 ERAS elements are currently implemented by a majority of cleft surgeons: avoiding prolonged perioperative fasting (67.7%), using hypothermia prevention measures (74.2%), and minimizing use of opioids (62.5%). A large majority of respondents noted they never administer bolus (71.0%) or infusion (80.6%) dosing of tranexamic acid; most of these surgeons also indicated that administering tranexamic acid “would not be a valuable addition” (67.7% and 71.0%, respectively). Short-acting sedatives are used by 12.9% and by 16.1% of surgeons in all patients during extubation and postoperative recovery, respectively. By contrast, 22.6% never use such agents during extubation and 48.4% never use it during postoperative recovery. Overall, 67.7% of respondents replied that they would be willing to implement an ERAS protocol for cleft palate repair.

**Conclusions:**

Many respondents report using interventions compatible with an ERAS approach and the majority are willing to implement an ERAS protocol for cleft palate repair.

## Introduction

Enhanced Recovery After Surgery (ERAS) protocols improve perioperative care for surgical patients by maintaining homeostasis and limiting stress. This enhances postoperative recovery, decreases postoperative morbidity, and reduces overall cost.^1,2^ General principles of ERAS include perioperative patient education, reduced preoperative fasting, minimizing perioperative opioid analgesia, minimally invasive techniques, early postoperative feeding and mobilization, minimizing use of catheters and drains, and auditing patient outcomes.^3^ ERAS protocols designed for specific procedures have been successfully implemented for adult patients with evidence of significant decrease in postoperative complications, length of stay (LOS), and overall cost.^3–5^

While ERAS protocols are well established for adult surgical procedures, their use in pediatric surgery is far more limited. As ERAS protocols were originally intended for adults, not all elements are useful or practical in the pediatric population. In response to increased interest in enhanced recovery for pediatric surgery, an expert panel comprised of pediatric surgeons, pediatric anesthesiologists, pediatric gastroenterologists, patient representatives, and nurse practitioners reviewed adult ERAS elements and selected 19 key elements that would be appropriate for children.^6^ Thus far, existing reports suggest that enhanced recovery programs in children significantly improve clinical outcomes, patient/family satisfaction, and cost-effectiveness.^7,8^ Many individual elements included in ERAS protocols are regularly utilized by pediatric surgeons outside of the context of a standardized protocol.^6^

Cleft palate repair involves meticulous repositioning of palatal tissues and velar musculature to repair the defect and begin restoring normal speech. Cleft palate repair could be a particularly useful context for implementation of a pediatric ERAS protocol because decreasing strain on the soft palate muscle repair can improve healing and protect its integrity, perhaps impacting future speech outcomes. The focus of this study is on the consensus and applicability of ERAS elements for primary cleft palate repair, as well as cleft palate surgeons’ familiarity and willingness to implement an enhanced recovery program in their individual practices. The purpose of this study, therefore, is to evaluate opinions and attitudes of academic cleft surgeons regarding implementation of ERAS elements for primary palatoplasty. We aimed to ascertain interest and obstacles for widespread implementation as well as inform our own cleft palate ERAS protocol.

## Methods

A closed-ended survey was created and administered electronically through REDCap, a secure data collection and analysis application.^9^ Survey data were collected and managed via REDCap electronic data capture tools and this study was deemed exempt for review by our institution's review board.

The survey's purpose and link were electronically mailed by the senior author to all 102 cleft/craniofacial surgeons practicing cleft surgery at an academic plastic surgery division/department in the United States. Demographic information was collected regarding primary area of practice, number of annual cleft palate repairs, number of cleft palate repairs performed in the previous year, and years of practice. Respondents were asked if they currently use a standardized ERAS protocol for cleft palate repair. To assess surgeons’ ERAS knowledge, respondents were asked to rate their overall knowledge of ERAS protocols and if implementing a protocol for cleft palate surgery would be a valuable addition to their clinical practice.

ERAS elements were selected from our own hypotheses and elements previously identified as pertinent for pediatric surgery in the literature.^6^ Several elements in the literature were not applicable to cleft palate repair: avoiding placement of nasogastric tube, no intraperitoneal or perianastomotic drains, early mobilization, early removal of urinary catheters, minimally invasive technique, and prevention of postoperative ileus. Given our infant patient population, early mobilization and early removal of urinary catheters were not applicable. A number of ERAS elements identified from the literature were developed in the context of surgery for the treatment of inflammatory bowel disease; therefore, those elements related specific to postoperative bowel management were excluded from our survey. Other ERAS elements from the literature that were either already standard of care or not pertinent for this age group included preoperative ERAS education, optimization of medical comorbidities, pre-incision antibiotic prophylaxis, and venous thromboembolism prophylaxis. In place of the intraoperative element “standard anesthetic protocol,” intraoperative and postoperative use of short-acting anesthetics, such as dexmedetomidine (DEX), was incorporated due to its sympatholytic effects and lack of respiratory depressive effects. Intraoperative infusion and bolus of tranexamic acid (TXA) were included as elements in the survey due to its antifibrinolytic and anti-inflammatory effects. All 12 elements included in the survey are listed in Tables 1 and 2.

**Table 1.** Surgeon Use of Each Individual Enhanced Recovery After Surgery (ERAS) Element in Cleft Palate Repair.

ERAS elements: current use	All patients	Most patients	Some patients	Never
*Preoperative*				
Nutritional screening	10 (32.2%)	6 (19.4%)	3 (9.7%)	12 (38.7%)
Avoid prolonged perioperative fasting	21 (67.7%)	2 (6.5%)	2 (6.5%)	6 (19.4%)
Administer non-opioid analgesia	10 (32.3%)	9 (29.0%)	8 (25.8%)	4 (12.9%)
*Intraoperative*				
Use hypothermia prevention measures	23 (74.2%)	2 (6.5%)	0 (0%)	6 (19.4%)
Use standardized protocol for maintaining normovolemia	14 (45.2%)	9 (29.0%)	4 (12.9%)	4 (12.9%)
Apply a multi-modal approach to post-operative nausea and vomiting	14 (45.2%)	11 (35.5%)	3 (9.7%)	3 (9.7%)
Use short-acting anesthetic agents	4 (12.9%)	9 (29.0%)	11 (35.5%)	7 (22.6%)
Administer a bolus of tranexamic acid	1 (3.2%)	3 (9.7%)	5 (16.1%)	22 (71.0%)
Administer infusion dosing of tranexamic acid	1 (3.2%)	3 (9.7%)	2 (6.5%)	25 (80.6%)
*Postoperative*				
Use short-acting anesthetic agents	5 (16.1%)	3 (9.7%)	8 (25.8%)	15 (48.4%)
Use goal directed fluid therapy model or zero fluid balance model to guide postoperative fluid management	6 (19.4%)	5 (16.1%)	5 (16.1%)	15 (48.4%)
Minimize use of opioids for postoperative pain control	18 (62.5%)	3 (9.7%)	10 (32.2%)	0 (0%)
Audit or review information on compliance and patient outcomes	9 (29.0%)	1 (3.2%)	1 (3.2%)	20 (64.5%)

**Table 2.** Responses to Whether Individual Enhanced Recovery After Surgery (ERAS) Elements Would be a Valuable Addition for Cleft Palate Repair.

ERAS elements: valuable addition to practice	Yes	No
*Preoperative*		
Nutritional screening	10 (62.5%)	6 (37.5%)
Avoid prolonged perioperative fasting	12 (75.0%)	4 (25.0%)
Administer non-opioid analgesia	13 (81.3%)	3 (18.8%)
*Intraoperative*		
Use hypothermia prevention measures	12 (75.0%)	4 (25.0%)
Use standardized protocol for maintaining normovolemia	11 (68.8%)	5 (31.3%)
Apply a multi-modal approach to post-operative nausea and vomiting	13 (81.3%)	3 (18.8%)
Use short-acting anesthetic agents	12 (75.0%)	4 (25.0%)
Administer a bolus of tranexamic acid	3 (18.8%)	13 (81.3%)
Administer infusion dosing of tranexamic acid	3 (18.8%)	13 (81.3%)
*Postoperative*		
Use short-acting anesthetic agents	7 (43.8%)	9 (56.3%)
Use goal directed fluid therapy model or zero fluid balance model to guide postoperative fluid management	9 (56.3%)	7 (43.8%)
Minimize use of opioids for postoperative pain control	13 (81.3%)	3 (18.8%)
Audit or review information on compliance and patient outcomes	12 (75.0%)	4 (25.0%)

Surgeons were queried regarding their use and familiarity with each element and if the element would be a valuable addition to their practice. Usage was rated on a 4-point scale: 1 representing “I use this element with all patients,” 2 representing “I use this element with most, but not all, patients,” 3 representing “I use this element with some, but not most, patients,” and 4 representing “I never use this element.” Knowledge about each ERAS element was rated on a 4-point scale: 1 representing “very knowledgeable about,” 2 representing “knowledgeable about,” 3 representing “somewhat knowledgeable about,” and 4 representing “not knowledgeable about.” Respondents were also asked to indicate whether each element would be a valuable addition to their practice. Lastly, the final question provided the opportunity for participants to describe any interventions they use to enhance recovery after surgery that were not included in the survey or provide additional comments.

Descriptive and inferential statistics were generated using REDCap and Microsoft Excel. Individual surgeon demographics were categorized into groups where appropriate. ANOVA one-way tests were used to assess possible associations between surgeon demographics (years in practice, practice composition, number of annual cleft palates repaired, number repaired in past year) and surgeons’ use, knowledge, and willingness to implement ERAS elements.

## Results

Of the 102 academic cleft palate surgeons surveyed, 31 completed the survey (30.4% response rate). Length of practice varied but 74.2% of respondents described their primary practice as pediatric craniofacial surgery (Table 3). 51.6% repair more than 20 cleft palates annually and 53.1% repaired more than 20 cleft palates the prior year. A majority (67.7%) rated they are “very knowledgeable about,” “knowledgeable about,” or “somewhat knowledgeable about” ERAS protocols in general. However, 61.3% “never use” a standardized ERAS protocol for cleft palate surgery. Overall, 68.8% indicated that a standardized ERAS protocol for cleft palate repair would be a valuable addition to their practice.

**Table 3.** Surgeon Demographics, Practice Composition, and Use, Knowledge, and Willingness to Implement a Standardized Enhanced Recovery After Surgery (ERAS) Protocol.

*Primary area of practice*	
General plastic surgery	5 (15.6%)
Pediatric craniofacial surgery	24 (75.0%)
Other	3 (9.4%)
*Annual number of cleft palate repairs*	
Less than 5	0 (0%)
5-10	4 (12.5%)
11-20	11 (34.4%)
Greater than 20	17 (53.1%)
*Number of cleft palate repairs performed in the last year*	
Less than 5	1 (3.1%)
5-10	4 (12.5%)
10-20	10 (31.3%)
Greater than 20	17 (53.1%)
*Years in practice*	
Less than 5	9 (28.1%)
5-9	5 (15.6%)
10-14	6 (18.8%)
15-19	5 (15.6%)
20-24	6 (18.8%)
Greater than 25	1 (3.1%)
*Current standardized ERAS protocol use*	
Use with all patients	10 (31.3%)
Use with most, but not all, patients	2 (6.3%)
Use with some, but not most, patients	1 (3.1%)
Never use this element	19 (59.4%)
*Knowledge of standardized ERAS protocol*	
Very knowledgeable	10 (31.3%)
Knowledgeable	3 (9.4%)
Somewhat knowledgeable	9 (28.1%)
Not knowledgeable	10 (31.3%)
*ERAS standardized protocol would be a valuable addition to practice*	
Yes	22 (68.8%)
No	10 (31.3%)

Tables 1 and 2 provide a summary of the respondents’ answers regarding each of the 12 ERAS elements surveyed. Three ERAS elements are currently used by a majority of surgeons for all patients: avoiding prolonged perioperative fasting (67.7%), using hypothermia prevention measures (74.2%), and minimizing use of opioids for postoperative pain control (62.5%). When surveyed about maintaining normovolemia and a multimodal approach to reducing post-operative nausea/vomiting, 46.9% reported using a standardized protocol with all patients but the vast majority rated that protocols for these elements would be a valuable addition to their clinical practice (84.4% and 90.6%, respectively). A large majority of respondents noted they never administer bolus (71.0%) or infusion (80.6%) dosing of TXA; most of these surgeons also indicated that administering bolus or infusion dosing of TXA “would not be a valuable addition to [their] practice” (67.7% and 71.0%, respectively). Short-acting sedatives such as DEX are used by 12.9% of surgeons for all patients during extubation and by 16.1% of surgeons for all patients during postoperative recovery. By contrast, 22.6% never use such agents during extubation and 48.4% never use it during postoperative recovery. More than half of survey takers (64.5%) do not audit information on patient outcomes but 83.9% stated that conducting such audits would be a “valuable addition to their practice.”

Practice composition, years in practice, annual frequency of cleft palate repair, and frequency of cleft palates repaired in the past year were analyzed to assess association with respondents’ use, knowledge, and willingness to implement a standardized ERAS protocol as well as each individual ERAS element. Practice composition was categorized as either pediatric craniofacial surgery, pediatric and adult craniofacial surgery, or general plastic surgery. Years in practice were categorized into 6 groups: less than 5 years, 5 to 9 years, 10 to 14 years, 15 to 19 years, 20 to 24 years, and 25 or more years. Annual number of cleft palate repairs and number performed in the last year were organized into 4 groups: less than 5, 5 to 10, 11 to 20, and greater than 20. On one-way ANOVA, there was no association identified between practice composition (*F* = 0.90, *P* = .44), years in practice (*F* = 1.56, *P* = .17), or annual frequency of cleft palates repaired (*F* = 0.41, *P* = .65) and use, knowledge, or willingness to implement ERAS elements. For number of cleft palate repairs performed in the last year, initial one-way ANOVA demonstrated a significant difference (*F* = 5.48, *P* = .001). However, an outlier group was identified (*n* = 1 for surgeon performing less than 5 cleft palate repairs in the past year). When re-grouping this outlier, there was no significant difference between or within groups (*F* = 0.36, *P* = .70).

The final questions in our survey solicited additional aspects of care that should be considered for a cleft palate ERAS protocol and provided an opportunity for additional commentary. Suggested additional ERAS components included gabapentin administration pre- and post-operatively, bupivacaine nerve blocks, early removal of any tongue traction suture, and frequent breaks of the Dingman oral retractor during the procedure. One respondent reported that there is no established protocol implemented at their institution for pediatric patients, but they are attempting to adapt it for the procedure. There was some skepticism about the necessity of the ERAS protocol and the utility of the survey. A few themes surfaced from the final question and are outlined in Table 4.

**Table 4.** Themes That Arose From the Open-Ended Question With Associated Quotations From Respondents.

Theme	Associated comments
Skepticism of ERAS necessity	“It's just a cleft palate. If your fistula or complication rate is too high, maybe you should find another way to make a living.”
Current ERAS implementation	“We don't formally use it but are trying to employ it. We use it for adults but hasn't been adopted fully for the peds population (the preop NPO instructions)”
Criticism of survey format	“…The way the questions are framed I think you are going to generate a lot of “noise” and internally inconsistent results. It's also unclear what the usefulness of the granularity of these questions is, as endeavors to promote it should cover all the elements of ERAS.”

## Discussion

Although numerous ERAS studies have demonstrated significant improvements in perioperative care for various surgical procedures in adults, evaluation of ERAS protocols in the pediatric literature is far more limited. In the past, there have been concerns that adult enhanced recovery elements may not pertain to children or may necessitate modification to better address the unique needs of pediatric patients. Despite this prior hesitation, 68.8% of survey respondents indicated they would be willing to implement an ERAS protocol and 31.3% already use a standardized protocol for cleft palate surgery. When asked about individual elements, a majority (56.3%-81.3%) of respondents indicated that they would be willing to implement 9 of the 12 ERAS elements surveyed. These elements consist of (1) nutritional screening, (2) avoiding prolonged perioperative fasting, (3) administering preoperative nonopioid analgesia, (4) using hypothermia prevention measures, (5) using a standardized protocol for maintaining normovolemia, (6) applying a multi-modal approach to postoperative nausea and vomiting, (7) using short-acting anesthetic agents (such as DEX) intraoperatively, (8) using goal directed fluid therapy model to guide postoperative management, and (9) audit information on compliance and patient outcomes. The remaining 3 elements were less accepted, indicating surgeon hesitation about their use in children or for cleft palate repair.

The following 3 ERAS elements were less accepted: (1) administering an intraoperative bolus of TXA, (2) administering an intraoperative infusion of TXA, and (3) using short-acting anesthetic agents (such as DEX) postoperatively. 71.0% and 80.6% of respondents never use a bolus or infusion of TXA intraoperatively during cleft palate repair. Additionally, 81.3% indicated that a bolus or infusion of TXA would not be a valuable addition to their practice. TXA is an antifibrinolytic agent which functions by competitively inhibiting the conversion of plasminogen to plasmin, therefore preventing degradation of fibrin clots by plasmin.^10^ Additionally, TXA has anti-inflammatory effects: plasmin activates and upregulates an inflammatory cascade by activating proinflammatory cells and gene expression.^11^ TXA's utility in plastic surgery is well-documented. It reduces postoperative blood loss and risk of hematoma, thereby reducing hospital LOS and need for postoperative blood transfusion.^10^ A postoperative hematoma after cleft palate repair can lead to a return to the operating room, wound dehiscence, infection, and/or atypical scarring.^10^ TXA's use across all surgical specialties is increasing significantly. It has been shown to reduce blood loss in trauma surgery,^12^ oral surgery,^13^ cardiac surgery,^13^ hepatic surgery,^14^ and has been shown to minimize pain and reduce drain output in orthopedic surgery.^15^ Adverse events such as thromboembolic events secondary to TXA are uncommon,^11^ but use has been associated with nausea, vomiting, and diarrhea.^10^ These side effects need to be considered carefully prior to inclusion in an ERAS protocol and patients should be monitored for such symptoms throughout the perioperative period. Still, due to TXA's antifibrinolytic and anti-inflammatory properties, as well as its promising data across most surgical specialties, it is an attractive element to include in an ERAS protocol for cleft palate repair.

Postoperative DEX use was the other less-adopted element in our survey. 48.4% of respondents never use it postoperatively and 56.3% of respondents rated that it would not be a valuable addition to their practice. Interestingly, however, 12.9% of survey respondents indicated they utilize it for all cleft palate repairs during extubation and 16.1% stated they utilize it during postoperative recovery. DEX is a highly selective α _2_-adrenergic agonist that inhibits release of norepinephrine, thereby inhibiting sympathetic activity and providing analgesia. With its short half-life and lack of respiratory depression, it is a preferred sedative in the post-anesthesia care unit (PACU). The most common perioperative complication of primary cleft palate repair is respiratory in nature,^16^ representing a potentially useful context for DEX utilization. DEX has been shown to ameliorate postoperative cognitive dysfunction^17^ and be more effective than propofol in treating emergence delirium in the postanesthetic stage of pediatric patients.^18^ It has also been associated with lower pain scores, decreased opiate use, and shorter hospital LOS in children who underwent decompression of a Chiari malformation.^19^ One potential limitation of using DEX is its higher cost compared to opioid analgesics, as the average daily drug cost of DEX is $452 compared to $230 for propofol or $126.30 for midazolam.^20^ But, it is possible that in the future the price of DEX will mimic the trajectory of midazolam, a historically costly drug but now is the favored benzodiazepine for ICU sedation at generic pricing.^21^ Because of its numerous benefits, DEX is an attractive element to include in an ERAS protocol for cleft palate repair. With its frequent utilization in a subset of respondents, it is possible that further familiarity and study of using DEX might increase usage among craniofacial surgeons during cleft palate repair.

This survey is a first step in developing an ERAS protocol specific to cleft palate repair. As mentioned previously, a cleft palate repair poses a unique opportunity for further optimization. While many procedures benefit from a standardized enhanced recovery program, reducing crying and strain on a fresh cleft palate repair make this procedure a particularly powerful context in which to consider application of a standardized ERAS protocol. Primary palatoplasty has a perioperative complication rate of 2.8%, 30-day fistula rate of 0.5%, and readmission rate of 1.9%.^16^ While these are relatively low complication rates, we hypothesize that perioperative optimization could improve longer-term speech outcomes in the setting of cleft palate repair. Our findings provide a starting point for pilot studies examining enhanced recovery from cleft palate repair. We used these results to formalize an ERAS protocol for cleft palate repairs at our children's hospital. This was designed in consultation with our anesthesia and nursing colleagues to ensure smooth implementation during the entire perioperative period. Our protocol includes the 9 elements designated by a majority of respondents as a valuable addition to their practice as well as the use of TXA and DEX (Table 5). We are prospectively studying perioperative and future speech outcomes in this cohort. To date, 24 patients have undergone palatoplasty in compliance with our ERAS protocol; none had a short-term symptom or complication related to use of TXA or DEX.

**Table 5.** Proposed ERAS Protocol for Cleft Palate Repair.

*Preoperative: before incision*
– Administer antibiotic – Decadron 0.5 mg/kg × 1 – Tranexamic acid (TXA) 30 mg/kg bolus × 1
*Intraoperative: After incision, prior to final closure*
– Tranexamic acid (TXA) 10 mg/kg/h – Minimize narcotic analgesia
*Extubation: after final closure, prior to extubation*
– IV Acetaminophen 15 mg/kg × 1 – Dexmedetomidine bolus titrated to effect and begin drip at 1 mcg/kg/h – Minimize narcotic analgesia
*PACU: after OR, prior to transfer to floor*
– Continue dexmedetomidine drip × 1 h (at least): Titrate drip to SBS score of 0, increasing dose by 0.25 mcg/kg/h q15 min Remain on drip for 1 h after reaching SBS score of 0 Wean drip to off by decreasing dose by half q20 min to 0.5 mcg/kg/h, remain at 0.5 mcg/kg/h for 20 min then discontinue drip Once drip is off, patient remains in PACU for 20 min prior to transfer to the floor – Start IV Toradol 0.5 mg/kg q8 h × 3 doses, first dose in PACU – Discontinue TXA 1 h after arrival to PACU – Minimize narcotic analgesia – Euvolemia – Clear liquid or breast milk diet once tongue stitch is removed
*Floor: after PACU, prior to discharge*
– Complete IV Toradol 0.5 mg/kg q8 h × 3 doses total – PO Acetaminophen ATC starting 6 h after intubation – Minimize narcotic analgesia – Euvolemia – Full liquid diet

While ERAS protocols specific to the pediatric population are increasing in the literature,^22,23^ obstacles to widespread implementation are likely. With 31.3% of respondents indicating they were “not knowledgeable” with enhanced recovery programs prior to this survey, further education is needed even among academic surgeons. Additionally, there was some skepticism raised by survey respondents about the necessity of an ERAS protocol for cleft palate repair in the absence of high complication rates. In addition to education, therefore, a belief in continual quality improvement is also required for ERAS adoption. However, procedure-specific, pediatric ERAS protocols are becoming more frequent, with recent publications drawing attention to the potential for quality improvement in this age group.^24–26^ One significant obstacle to successful implementation of an ERAS protocol is the necessity of multidisciplinary buy-in. Administrators, anesthesia providers, nurses, and surgeons must work together to develop, support, and continually better such protocols to optimize patient results. In our practice, we have facilitated this cooperation by soliciting feedback from all stakeholders (preop nurses, anesthesia, PACU nurses, floor nurses, and parents). Regularly scheduled interdisciplinary meetings are useful for reviewing the protocol, addressing concerns, and monitoring study progress.

It should be acknowledged that most of the individual interventions incorporated in an enhanced recovery pathway are well-established in the pediatric setting. There are many studies demonstrating the safety and efficacy of these individual ERAS elements in children.^7,27–31^ As survey responses showed, some surgeons are already safely utilizing a standardized protocol or specific ERAS elements. Next steps involve efforts to increase awareness of literature regarding pediatric ERAS components and ways to ease implementation to develop a standardized approach to cleft palate repair. Evidence demonstrating the benefits of standardized care have been well-documented in the literature.^32^ Since adult ERAS procedures have demonstrated decreased LOS, decreased total procedure-related morbidity,^33^ decreased post-operative pain scores, and decreased complication rates,^34^ the potential in the pediatric population is extremely promising. The perioperative period is an emotionally challenging time for families, who often report feeling unprepared for surgery, postoperative pain, and recovery.^35^ An ERAS protocol can significantly improve patient care and the family's overall perioperative experience.

Our study has notable limitations. Our survey response rate was 30.4%, and those who did not complete the survey may differ in their use, knowledge, and willingness to implement ERAS elements. The study's response rate is comparable to APSA survey results that have been published.^36,37^ Surveys are prone to self-selection bias, and it is possible that the respondents who were willing to participate in the survey are more knowledgeable or open to ERAS protocols. Our survey targeted only academic craniofacial surgeons, but the demographics of respondents were heterogeneous with varying years of practice, number of cleft palate repairs performed, and status of current ERAS adoption for cleft palate repair. Therefore, this sample might accurately represent the practice of academic craniofacial surgeons who treat cleft palate across the country. Another limitation is that some surgeons might not be aware of all the medications administered during palatoplasty, thereby limiting survey responses. Our survey did inquire if the surgeon or the anesthesia team administers the ERAS elements to prompt respondents to consider the role of their anesthesiologists. The survey also asks about the respondents’ knowledge of administration of each element and has an open response section at the end where they could indicate any uncertainty. Finally, with any survey, responses are subject to recall bias. It should be considered that respondents could have completed the survey with a particular case in mind instead of considering their institution or group's practice. However, if focused on a particular case, the case could have had a good or bad outcome and responses may have been affected by such experience.

## Conclusions

Many respondents report using interventions which are consistent with an ERAS approach to perioperative care, and the majority would be willing to implement a standardized ERAS protocol for cleft palate repair. Use of TXA and DEX was uncommon and unlikely to be quickly adopted. Prospective studies are underway to evaluate perioperative and speech outcomes after interdisciplinary adoption of an ERAS protocol for cleft palate repair.
